# Supracerebellar transtentorial approach to the parahippocampal gyrus

**DOI:** 10.3171/2024.4.FOCVID2455

**Published:** 2024-07-01

**Authors:** Pavel Pichardo-Rojas, Arjun Suresh Chandran, Fernando De Nigris Vasconcellos, Ellery Wheeler, Ryan McCormack, Nitin Tandon

**Affiliations:** Vivian L. Smith Department of Neurosurgery, UTHealth Houston, Texas

**Keywords:** transtentorial approach, supracerebellar, parahippocampal gyrus, cavernous malformation

## Abstract

The supracerebellar transtentorial technique (SCTT) is a versatile approach that grants access to medial and basal temporal (MBT) regions without transgressing normal lateral cortex, damaging the hippocampus, or requiring significant brain retraction. This video illustrates the SCTT in resecting a cavernous malformation within the parahippocampal gyrus to alleviate associated epilepsy and preserve cognition. The authors outline the anatomical considerations, alternative approaches, positioning, craniotomy, and dural opening. They demonstrate how to access the supracerebellar space, elevate the dura toward the tentorial incisura, and resect the malformation. This video serves as a practical reference for management of MBT lesions via minimally invasive procedures.

The video can be found here: https://stream.cadmore.media/r10.3171/2024.4.FOCVID2455

## Transcript

This presentation is about a supracerebellar transtentorial approach to a lesion in the parahippocampal gyrus, an approach that enables us to get to the abnormality with very little consequence to normal functional temporal lobe tissue.^1–7^

### 0:37 Case Presentation.

In this particular instance, we had a 23-year-old right-handed gentleman who had a seizure while driving and then subsequently another grand mal tonic-clonic seizure. He is trained as an engineer and is working as one, and was extremely concerned about the impact of any intervention on his cognitive abilities, as well as the ability to keep his job and his high level of functioning. He had no other prior relevant history and has been on anticonvulsants since those episodes. He underwent a video-EEG study for an interval of 23 hours, which revealed several interictal abnormalities in the right temporal lobe but no seizures were captured in that interval period. The patient was found to have a high IQ, and given all of these factors, we were driven to make a decision to minimize the impact of our intervention on his cognitive function.

### 1:32 Brain MR Imaging Review.

So you can see here a coronal MRI scan on the left which shows an area of abnormality in the parahippocampal gyrus with the hippocampus just above it, and a large amount of hemosiderin staining in this location. Multiple bubbles of hemorrhage on the right-hand side, which are high intensity on noncontrasted T1-weighted sequences suggestive of relatively recent hemorrhage. This is the contrasted MRI scan that shows the same findings and a FLAIR sequence that shows the blooming artifact. This is a small clip through his coronal oblique T2-weighted MRI scans that show the extent of this lesion, the multiple sites of hemorrhage, the areas of associated cystic change related to this malformation, and then the same is visible here on a sagittal contrasted MRI scan.

### 2:27 Potential Surgical Approaches.

So obviously there are several ways of dealing with this problem. One would be a traditional anterior temporal lobe resection (ATL), which would sacrifice the temporal pole and likely sacrifice a substantial portion of the uncus.

The other approach would be through a transsylvian approach that would expose the insula and then from the inferior circular sulcus of the insula, a trajectory to the ventricle. The problem with this approach is that the hippocampus is sort of on the way to the lesion. It would either need to be significantly mobilized or resected to get access to the lesion itself. In addition, the temporal stem would be cut in some portions, which is likely to have its own cognitive impact.

A third approach could be a subtemporal approach, very reasonable to do in this circumstance, but given the significant posterior extent of this lesion is likely to be limited by the tethering effect of the vein of Labbé and its relationship and attachment to the sigmoid sinus.

Another approach to this lesion could be a transtemporal approach to the middle temporal gyrus, as advocated in the past by Olivier.

Of course, laser ablation in this day and age is a very reasonable option for these lesions, but the profuse amount of hemosiderin staining, the multiple areas of hemorrhage and cystic changes associated with that, make it unlikely that that would be a viable alternative.

So we elected to do this, which is a supracerebellar transtentorial approach. It’s a bit of a long approach. The posterior extent of the lesion was about 7 cm in the front end with about 9 cm from the back of the cerebellum.

### 4:03 Patient Positioning, Incision, and Craniotomy.

This is how the patient was positioned, three-quarters prone with the right side up. He had also undergone placement of a lumbar drain that we used to facilitate retraction. These are the steps that we undertook, which we will discuss in more detail with the surgical video. With that position, here is the approximate incision. The two slot-like holes made on the occipital bone that enable a craniotomy that straddles the transverse sinus and extends both above and below it.

The exposure of the posterior fossa dura, which is then opened in a V-shaped fashion retracted superiorly, slightly tenting up the tentorium as well as the transverse sinus, while not occluding it or creating any venous back pressure, but enabling a good exposure of the tentorium, which is then exposed and cut.

### 4:56 Initial Dissection and Tentorial Exposure.

This is the early step in exposing the cerebellum and exposing the incisura, the tentorial edge, opening the arachnoid over the ambient cistern and exposing the posterior cerebral artery.

### 5:16 Tentorial Incision.

The tentorium is then explored and I use a Bovie cautery to start making the cut in the tentorium, which is otherwise hard, and then using a curved microscissors to extend this cut toward the midline. This creates a V-shaped piece of tentorium that is freed, except in its posterior attachment and enables it to be mobilized. This is us cutting the last little bit of the medial aspect of the tentorium to expose the fusiform gyrus and the parahippoccampal gyrus above us. And then this is a little stitch that I place in the tentorium to tent it away and expose this posterior cerebral artery branch, in front of which is where we are going to make our corticectomy.

### 6:03 Parahippocampal Corticectomy.

So this is the coagulation of the posterior parahippoccampal gyrus and a small amount of leucotomy is performed through the most posterior extent of the parahippocampal gyrus right adjacent to where the cavernoma is. And in less than about 5 or 6 mm of this leucotomy being performed we enter a cystic space right there, which is hemosiderin stained and there is a large blister of blood products contained within that most recent hemorrhage of the cavernoma, which is then decompressed and drained, creating space to visualize the cavernoma, which I then mobilized away from each of its attachments.

### 6:45 Cavernoma Resection.

This is separating it from its basal attachment, its medial attachment, all the while working right at the intersection of the cavernoma and the surrounding gliotic white matter. Shrinking it slightly to enable a little more visualization, and now separating it from its anterior and superior attachments, which is where there was a very small developmental venous anomaly, as these lesions often do. Now here we are mobilizing the cavernoma laterally and separating it from its lateral attachments. There is some normal white matter, and there is hemosiderin-stained tissue just lateral to it that we coagulate and aspirate, all the while separating the cavernoma, mobilizing it into the cavity and away from the surrounding healthy brain tissue.

### 7:37 Cavernoma Removal and Cavity Exploration.

After it is completely separated, we deliver this cavernoma in a single piece. The rest of the cavity then is very carefully inspected and explored for any hemosiderin staining. That is the hippocampal hilus that we are looking at from below and all the vessels supplying the hippocampus are in there, which we elected to preserve. It would be not unreasonable in this case to sacrifice the hippocampus, but we had that conversation with the patient and his family, and he was very interested in saving it at all costs, and so we left the hippocampus and therefore also left those vessels to the hippocampus.

### 8:10 Closure.

The dura is then placed back to its original position, gradually and gently restoring that separation plane between the cerebellum and inferior temporal lobe. A small amount of Surgicel is placed on top of the cerebellum to prevent any delayed oozing. It is a matter now of closing the dura, which we did in this case with a piece of bovine pericardium and the bone plate. The skin was closed in the usual fashion.

### 8:38 Postoperative Course.

The patient did extremely well. He stayed in the hospital a couple of days and was discharged with no neurological consequences, and at visit in the office about 8 days after surgery, expressed that his memory by his estimation was actually better.

### 8:53 Similar Illustrative Case Presentations.

I am going to end by summarizing a few other cases that are similar to this. So here is an example of a 4-year-old girl, who had complex partial and generalized seizures with a lesion in her posterior parahippocampal gyrus and her fusiform gyrus, which was also approached via a supracerebellar transtentorial approach. Here we are opening the tentorium, coagulating it, reflecting it, and then putting in an electrode to record from the lesion, which showed abnormal discharges, and then the lesion was resected. Here is her pathology, which was a ganglioglioma which was associated with a cortical dysplasia, and here is her MRI scan at 6 months postoperative. She is now about 15 years postoperative and is seizure-free. We can see on this MRI scan that the hippocampus was floating above the cavity of the resection.

This is another example, this is a young woman in her 20s who presented with complex partial seizures and this lesion that is more posterior in her parahippocampal gyrus, around where it becomes the isthmus of the parahippocampal gyrus becoming the cingulate gyrus. This is also visible on FLAIR as well as on contrasted T1-weighted images. And here she is after her resection, also with a similar approach.

This illustrates the range of regions that you can access with this approach, and the safety of doing these procedures.

## Disclosures

The authors report no conflict of interest concerning the materials or methods used in this study or the findings specified in this publication.

## Author Contributions

Primary surgeon: Tandon. Assistant surgeon: Chandran, McCormack. Editing and drafting the video and abstract: Pichardo-Rojas, Chandran, De Nigris Vasconcellos, Wheeler. Critically revising the work: all authors. Reviewed submitted version of the work: all authors. Approved the final version of the work on behalf of all authors: Pichardo-Rojas. Supervision: Pichardo-Rojas, McCormack.

## Supplemental Information

### Patient Informed Consent

The supracerebellar transtentorial approach to the parahippocampal gyrus did not necessitate patient informed consent. This is because all videos and images utilized were part of the standard clinical care provided to the patient. No separate study was conducted, and all data were de-identified to ensure patient privacy and confidentiality. Therefore, the use of these materials did not involve any additional procedures or interventions beyond those required for routine medical treatment.
